# Who’s misbehaving? Perceptions of unprofessional social media use by medical students and faculty

**DOI:** 10.1186/s12909-016-0572-x

**Published:** 2016-02-18

**Authors:** Elizabeth A. Kitsis, Felise B. Milan, Hillel W. Cohen, Daniel Myers, Patrick Herron, Mimi McEvoy, Jacqueline Weingarten, Martha S. Grayson

**Affiliations:** Departments of Medicine, Albert Einstein College of Medicine, 1300 Morris Park Avenue, Bronx, NY 10461 USA; Epidemiology & Population Health, Albert Einstein College of Medicine, 1300 Morris Park Avenue, Bronx, NY 10461 USA; Family & Social Medicine, Albert Einstein College of Medicine, 1300 Morris Park Avenue, Bronx, NY 10461 USA; Psychiatry & Behavioral Sciences, Albert Einstein College of Medicine, 1300 Morris Park Avenue, Bronx, NY 10461 USA; Pediatrics, Albert Einstein College of Medicine, 1300 Morris Park Avenue, Bronx, NY 10461 USA

## Abstract

**Background:**

Social media use by physicians offers potential benefits but may also be associated with professionalism problems. The objectives of this study were: 1) to examine and compare characteristics of social media use by medical students and faculty; 2) to explore the scope of self- and peer-posting of unprofessional online content; and 3) to determine what actions were taken when unprofessional content was viewed.

**Methods:**

An anonymous, web-based survey was sent to medical students and faculty in October, 2013 at the Albert Einstein College of Medicine in Bronx, New York.

**Results:**

Three-quarters of medical students reported using social media “very frequently” (several times a day), whereas less than one-third of faculty did so (*p* < .001). Medical students reported using privacy settings more often than faculty (96.5 % v. 78.1 %, *p* < .001). Most medical students (94.2 %) and faculty (94.1 %) reported “never” or “occasionally” monitoring their online presence (*p* = 0.94). Medical students reported *self*-posting of profanity, depiction of intoxication, and sexually suggestive material more often than faculty (*p* < .001). Medical students and faculty both reported *peer*-posting of unprofessional content significantly more often than *self-*posting. There was no association between year of medical school and posting of unprofessional content.

**Conclusion:**

Medical students reported spending more time using social media and posting unprofessional content more often than did faculty.

**Electronic supplementary material:**

The online version of this article (doi:10.1186/s12909-016-0572-x) contains supplementary material, which is available to authorized users.

## Background

Widespread use of social media has impacted medicine in diverse ways. Recent systematic literature reviews highlight both the benefits and problems related to social media use in medicine [1, 2]. A variety of ethical issues may arise from social media use in the health care setting [3, 4], one of which is professionalism. Because of concerns about the risk of unprofessional behavior in using social media, several organizations have issued guidelines for the appropriate use of these platforms [5–7]. Yet awareness of and consensus with these guidelines is unclear, and online unprofessional behavior continues.

State medical boards reported that online violations of professionalism by physicians were quite common, and often led to disciplinary actions [8, 9]. An analysis of blogs written by health care professionals showed that confidential information about patients was revealed, and that the authors and the medical profession were depicted in negative ways [10].

In addition to physicians in practice, physicians in training have also been reported to post unprofessional content [11]. Because younger generations use social media more often [12] and may have not yet fully developed their professional identities, it is possible that medical students are at greater risk for online professionalism lapses than practicing physicians. Yet few comparisons of social media use by medical students and physicians are available. Differences in usage patterns and attitudes toward social media between students and faculty may be important. For example, physician educators are charged with teaching medical students about professionalism, including online professional behavior, but they may be less familiar with social media than the students for whom they are responsible.

The objectives of this study were to evaluate and compare social media use by medical students and medical school faculty. We also sought to assess and compare self-reports of medical student and faculty posting or viewing of unprofessional online content,, and to determine what actions were taken when unprofessional online content was observed.

## Methods

### Survey design

A 32-item core survey instrument regarding social media use was created based the existing literature, and input from focus groups done at our institution [11]. The instrument was modified for each of the target populations (medical students and faculty) based upon their differing characteristics, and the survey was piloted. Please see survey tools in Additional files 1 and 2. The Institutional Review Board of the Albert Einstein College of Medicine deemed the study exempt.

All medical students and faculty at the Albert Einstein College of Medicine were invited to complete the anonymous, web-based (SurveyMonkey) survey in October, 2013. The initial request for participation in the survey was sent by e-mail to all medical students and all faculty at the Albert Einstein College of Medicine College of Medicine (dedicated listservs) by the Senior Associate Dean for Medical Education. Follow-up requests were also sent by e-mail. No incentives for survey completion were offered.

The surveys collected information on participants’ gender and age. In addition, the medical student survey asked for year of medical school. The faculty survey asked respondents to identify their specialties. Medical students and faculty were asked about their familiarity, competence, and frequency of use of Facebook, YouTube, LinkedIn, Twitter, Google+, Pinterest and Instagram. Both groups were asked to rate the importance of certain factors in influencing their social media use as well as concerns they had about social media use. Participants were also asked about whether they monitored their online presence, either by Google search or Google Image search, and to indicate what actions they had taken (if any) if they discovered online information about themselves that they believed should not be publicly available. Medical students and faculty were asked whether they had posted unprofessional online content, and whether they had seen unprofessional content posted by classmates or colleagues, respectively. Examples provided of unprofessional online content were identifiable patient information, use of profanity, depiction of intoxication, and sexually suggestive material.

### Statistical analysis

Respondent characteristics were quantified by calculating frequencies. Comparisons of medical student and faculty responses were calculated by using the chi-square or Fisher’s exact test as statistic as appropriate. Likert scales were analyzed as ordinal variables with bivariate Spearman correlations because the differences between the ordered responses could not reasonably be assumed to be on an interval scale. For comparisons of proportions, we dichotomized the values combining the “positive” responses, i.e. “somewhat” and “very” and contrasted these to the non-positive (i.e. the neutral and negative responses). We were concerned that the subjective distinction between “somewhat” and “very” would be too variable among the participants and would likely differ in another sample whereas a positive vs. a non-positive response would likely be more robust and more generalizable to other samples as well as being simpler to interpret.

Denominators for students and faculty varied slightly because some medical student and faculty respondents did not answer every question. Percentages reported for the responses are the percentages of those who answered that question. A two-tailed alpha of 0.05 was used to denote statistical significance. Analyses were performed with SPSS for Windows (Version 20).

## Results

### Respondent characteristics

63.5 % (496/781) of medical students and 22.6 % (614/2713) of faculty members responded to the survey. A comparable proportion of medical student and faculty respondents were women (49.5 % v. 52.0 %, respectively; *p* = 0.55). 74.8 % of medical student respondents were between the ages of 21 and 26, and 74.1 % of faculty respondents were between the ages of 30 and 59. Comparable numbers of students from each year of medical school were represented. Internal medicine (including internal medicine subspecialties) was the most common specialty of the faculty respondents (19.5 %).

### Social media usage

34.1 % of medical student respondents reported using social media six or more hours per week, compared with 9.0 % of faculty respondents (*p* < .001). 15.9 % of medical student respondents reported frequency of social media use as “do not use” or “less than one hour” compared with 60.1 % of faculty respondents (*p* < .001).

**Fig. 1:**
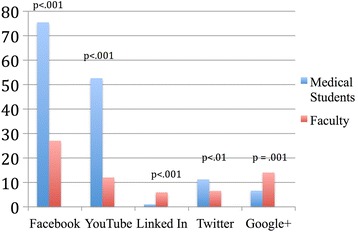
% respondents using social media platforms frequently* or very frequently**, *frequently = daily, **very frequently = several times per day

The majority of medical student respondents used Facebook frequently (defined as daily) or very frequently (several times a day) (Fig. 1). Medical students used Facebook, YouTube, and Twitter more often than faculty, and faculty used LinkedIn and Google + more often than medical students (Fig. 1). The majority of medical student and faculty respondents never used Pinterest, Tumblr and Instagram.

### Barriers to social media usage

Medical students (72.6 %) were more likely than faculty (59.7 %) to rate “concern about harm to professional image” as a somewhat or very important factor that prevents them from using social media (*p* < .001). 41.3 % of medical students rated lack of knowledge as a somewhat or very important factor that presents obstacles to social media use, compared with 51.7 % of faculty respondents (*p* = .001).

While most medical student and faculty respondents reported using privacy settings on social networking platforms, significantly more medical students reported using them (96.5 % v. 78.1 %, *p* < .001). Most medical student and faculty respondents reported never or occasionally monitoring their online presence by performing Google searches of themselves (94.2 % v. 94.1 %, *p* = 0.94) or checking Google Image for online photos of themselves (94.2 % v. 94.1 %, *p* = 0.94).

### Posting of unprofessional online content

Medical student respondents reported that they found their online presence to be “unprofessional” (21.8 %) or “absent” (24.0 %) more often than faculty respondents (5.5 % and 8.1 %, respectively (both *p* < .001)). More medical student than faculty respondents reported taking action if they found information online about themselves that they believed should not be publicly available, as shown in Table 1.

**Table 1:** Actions taken if respondents found information they believed should not be publicly available

Action	Medical students (%) 496 respondents	Faculty (%) 614 respondents	*P* value*
Deleted people from my “friends” list	176 (35.5)	84 (13.7)	<.001
Deleted comments made by others on my profile	264 (53.2)	88 (14.3)	<.001
Removed my name from photos that were tagged to identify me	319 (64.3)	114 (18.6)	<.001
I have not taken action	68 (13.7)	271 (44.1)	<.001

*P value calculated from Pearson chi-square or Fisher’s exact test if any cell had an expected value < 5

**Fig. 2:**
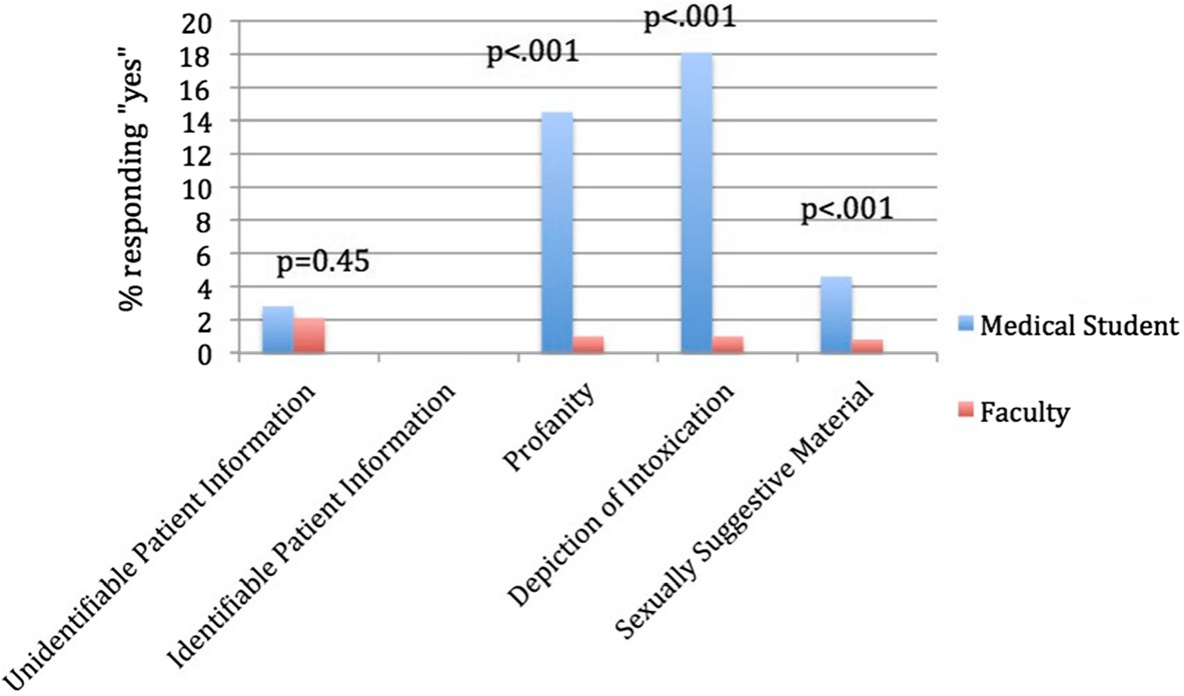
Response to the question, “Which of the following types of information have you posted online yourself?”

**Fig. 3:**
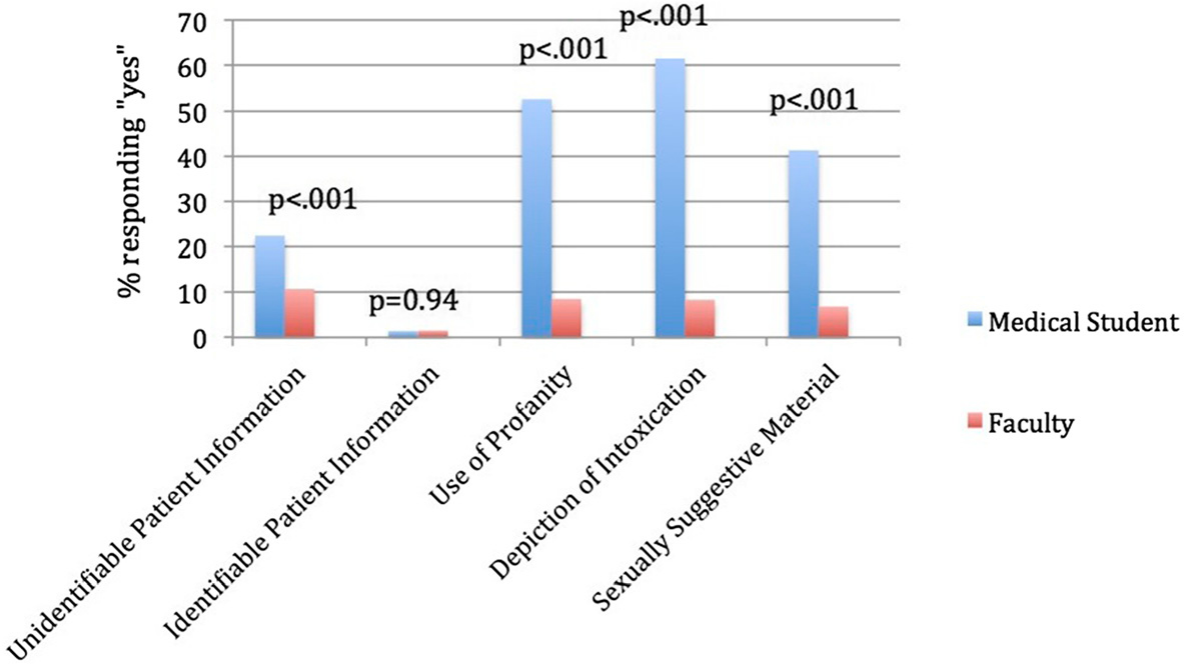
Response to the question, “Which of the following types of information have you seen posted online by a classmate or colleague?”

Viewing unprofessional content online posted by classmates or colleagues was reported more frequently by medical students and faculty than posting of such content themselves (*p* < .001). Medical student posting (Fig. 2) and viewing (Fig. 3) of unprofessional content online was reported significantly more often compared with faculty respondents. There was no association between year in medical school and posting of unprofessional content (data not shown). Both groups reported no instances of self-posting identifiable patient information. Two medical student and two faculty respondents reported having observed classmates/colleagues posting identifiable patient information.

Almost all medical students (92.4 %) and the majority of faculty (83.4 %) rated violations of patient confidentiality as somewhat or very important concerns about social media use (*p* < .001). Similar proportions of medical students (91.1 %) and faculty (83.5 %) rated posting inaccurate medical information for patients as somewhat or very important concerns. Most medical student respondents (92.5 %) ranked public perceptions of unprofessional behavior by them as a somewhat or very important concern about social media use, compared with 69.6 % of faculty (*p* < .001). 89.0 % of medical students ranked public perceptions of unprofessional behavior by colleagues as a somewhat or very important concern about social media use, compared with 66.5 % of faculty (*p* < .001).

Respondents were given the opportunity to provide comments, some of which identified additional social media content that was felt to be unprofessional, including the following:

- “Material that is derogatory towards certain types of patients (i.e. low income patients/Medicaid patients)
- Remarks disparaging of professors
- Comments about work…either about colleagues or patients or work stress
- Political contributions”

### Correlation of age and social media platform

Older age was inversely correlated with frequency of Facebook, YouTube, Instagram, Pinterest, Twitter, Tumblr and LinkedIn use for faculty respondents (*r* = -0.37, -0.26, -0.23, -0.21, -0.18, -0.14, -0.08, respectively). All differences were statistically significant except for LinkedIn.

## Discussion

Social media use has infiltrated society, and medicine is no exception. The results of the current survey are consistent with population studies showing that younger people use social media more often than older adults [12], and confirm previous reports that physicians in training use social media networks more commonly than faculty [13–16].

Our survey found that that over 95 % of medical students reported using privacy settings, and that they did so significantly more often than faculty. This finding contrasts with that of Landman et al [14], who reported that half of surgical house staff and faculty Facebook pages at a single university were publicly accessible, suggesting less frequent use of privacy settings. The latter study was published in 2010. Our data are consistent with a more recent study from the United Kingdom that found that 93 % of medical students report using privacy settings on Facebook [15], and another from Canada that actually searched the Facebook profiles of medical students, and reported that most students used privacy settings [16]. Perhaps with time and increasing educational interventions, students are becoming more aware of the importance of using privacy settings. The greater use of privacy settings on social networking platforms by medical students compared with faculty may be due to a lack of awareness of the importance of privacy settings among faculty or a lack of knowledge about how to use them.

This study highlights multiple factors that may contribute to the difference in pattern of social media use observed between medical students and faculty. One such factor is lack of knowledge, which was reported more often by faculty than students as an important reason for not using social media. Another reason health care professionals may shy away from social media use is concern about harm to professional image. Of note, while the majority of medical students and faculty indicated that concern about harm to their professional image was a factor that prevented them from using social media, medical students cited this concern more often than faculty. However, it is unclear that these concerns exist in all cultures. A survey of medical students’ attitudes about social media use in Turkey suggested that students are unaware of ethical concerns posed by social media use [17].

An interesting paradoxical observation from our study is that while students seemed more concerned than faculty about their professional images, their online behavior did not reflect this concern. Medical students reported that they considered their online presence to be unprofessional four times more often than faculty. In view of these findings, one might expect medical students to monitor their online presence regularly. Surprisingly, they reported self-monitoring rarely, and at a rate similar to faculty.

One student respondent commented that monitoring his online image “won’t be important until I have an [online] professional presence to monitor.” So perhaps students are aware that they will need to monitor their digital footprints, but don’t see the necessity to do so while they are in medical school. Or possibly students lack awareness of or agreement with guidelines that recommend that monitoring be done, or perhaps lack the knowledge regarding how to do it. Educational interventions may stimulate medical students’ plans to monitor and modify their online presence [18]. Such programs for faculty may have a similar effect. As a result of this study, we have incorporated a session on social media and professionalism into our required bioethics curriculum. It informs our medical students of existing social media guidelines, encourages them to monitor their digital footprints, and provides them with an opportunity to discuss a variety of real-life social media scenarios in which professionalism lapses may have occurred.

More students than faculty reported taking action if they found objectionable information about themselves online The inaction by faculty may be due to a lack of knowledge about how to handle such situations. Of concern, while faculty are generally responsible for educating students about what constitutes professional behavior, their lack of familiarity with social media and lack of responsiveness to acknowledged unprofessional postings raises questions about their expertise in carrying out this important educational activity. Indeed, Patel, et al. [19] suggested that residents are more appropriate social networking mentors for students than faculty, based on their more similar usage of and attitudes toward social media. Because of these issues, we have held several educational symposia for our entire medical school faculty on social media and professionalism.

Violation of confidentiality is one of the most concerning professionalism lapses in social media. A 2009 study found that 13 % of deans of student affairs reported incidents of violations of confidentiality by medical students [11]. In contrast, our study suggests that this problem occurs much more rarely, perhaps reflecting a greater sensitivity to confidentiality issues over time.

Posting of other unprofessional content online (e.g., profanity, depiction of intoxication, and sexually suggestive material) was observed in this study as well as the 2009 report. Our survey also found that medical students reported *observing peers* posting unprofessional content online more often than faculty, but less frequently than found by Osman, et al. (88 %) [15]. The observation that students reported posting unprofessional online content more often than faculty may reflect the more nascent professional formation of students, or perhaps a difference in perception of the types of behaviors that are permissible overall, or more specifically online [20, 21]. Generational differences in perspectives about what constitutes unprofessional behavior may account for these results. Qualitative research about differences in perceptions of unprofessional behavior could help confirm this speculation.

The relative anonymity, invisibility, and asynchronicity of the Internet may result in posting of content that would otherwise be kept private [22]. Concerns about this online disinhibition effect remain, at least with regard to medical students, who are generally at an age when they are still undergoing moral development. It has previously been reported that use of Facebook decreases as trainees move from residency to fellowship [23], suggesting a possible heightened sensitivity to problems associated with Facebook use as one advances in a professional career. We therefore hypothesized that medical student reports of posting online unprofessional content would decrease as the students progressed in medical school, reflecting an advance in their education and an increased awareness of the potential impact of their behavior on professionalism. However, this hypothesis was not supported by the data, which did not show an association between year in medical school and posting of unprofessional content. We can only speculate on the reasons for this. The decline in professionalism overall in medical school is a national phenomenon well documented in the literature, so as new modalities for communication take hold, one can assume that they would reflect the general attitudes and beliefs about professionalism. Our medical school created a curriculum on social media and professionalism to help prevent this from happening. Another possible explanation is that medical students are aware of the importance of online professionalism, but do not feel it is relevant to them until they graduate and have an actual online professional identity.

Self-reports of unprofessional online content were several-fold lower than reports of classmates’ and colleagues’ posting of unprofessional content. The reason for this discrepancy is unclear, but is concordant with the literature suggesting that physicians are poor at self-assessment [24]. Respondents may have denied to themselves that they posted unprofessional content, as the social undesirability of behaving in an unprofessional manner is very strong. Or maybe they held their colleagues to a higher standard. A simpler, albeit less likely explanation is that they all observed the same unprofessional posting by one classmate or colleague.

Professional identity formation among medical students now entails consideration by students about whether and how they can continue to use social media as physicians. According to Cruess, et al. [25], students may develop “identity dissonance” when components of their identity as physicians conflict with their identity as laypersons. As one of our medical student respondents commented in the survey, “Professionalism is necessary in medicine, but so is maintaining a good personal life, which social media can help to do.” Continued reports of unprofessional social media behavior may reflect the experience of the current medical student who struggles to develop a professional identity while continuing to maintain the type of active, online social life characteristic of the current generation.

This study has several limitations. Responder bias may be present, especially in the faculty sample. Lower levels of self-report than peer-report of unprofessional postings could be due to social desirability bias. The faculty response rate of 23 % was relatively low, but not atypical of web-based physician surveys [26]. The study was conducted at a single institution, so its generalizability to other institutions is unknown. Since the study was based upon a survey, it reflects the perceptions of the respondents, rather than the actual online behaviors of participants.

## Conclusions

Our survey shows that medical student posting of unprofessional material does not decrease during medical school, and that medical students self-post and notice peers’ unprofessional posts more often than do faculty. The data suggest that medical students and faculty may have different perceptions of social media professionalism, perhaps shifting the question from “Who is misbehaving?” to “What constitutes misbehaving?” Further research could help determine whether these differences disappear as students further progress through professional formation, or whether they represent an evolving generational disagreement in the definition of professional behavior.

### Consent for publication

Not applicable.

## Additional files

Additional file 1:
**Einstein Medical Student Survey on Social Media.** (PDF 437 kb) Additional file 2:
**Einstein Faculty Survey on Social Media.** (PDF 460 kb)
