# Supplementary material for: Who’s misbehaving? Perceptions of unprofessional social media use by medical students and faculty
Source: BMC Med Educ. 2016 Feb 18;16:67. doi: 10.1186/s12909-016-0572-x (PMC4757980; doi:10.1186/s12909-016-0572-x)
Supplement: Additional file 1: — Einstein Medical Student Survey on Social Media. (PDF 437 kb) [file 12909_2016_572_MOESM1_ESM.pdf]

EINSTEIN
Albert Einstein College of Medicine
OF YESHIVA UNIVERSITY

Einstein Medical Student Survey on Social Media

1. What is your gender?

- Male
- Female
- Other

2. What is your age?

- 21-23
- 24-26
- 27-29
- 30-32
- 33-35
- 36-40
- 41-49
- 50 or older

3. How do you identify yourself?

- White
- Black or African American
- American Indian or Alaska Native
- Asian Indian
- Pakistani
- Asian
- Native Hawaiian or other Pacific Islander
- Spanish/Hispanic/Latino/Latina

Other (please specify)

4. What year of medical school are you currently in?

- ◯ First
- ◯ Second
- ◯ Third
- ◯ Fourth

Other (please specify)

[ ]

5. How familiar are you with each of the following social media services?

	Never heard of it	Heard of it; not sure of its purpose	Familiar with its purpose	Very familiar with its purpose
Facebook	◯	◯	◯	◯
Twitter	◯	◯	◯	◯
Linked In	◯	◯	◯	◯
YouTube	◯	◯	◯	◯
Google+	◯	◯	◯	◯
Pinterest	◯	◯	◯	◯
Tumblr	◯	◯	◯	◯
Instagram	◯	◯	◯	◯

If there are other social media services with which you are familiar, please list and rate your familiarity here.

[ ]

6. How competent are you at using each of the following social media services?

	Not at all	Beginner	Competent	Highly competent
Facebook	○	○	○	○
Twitter	○	○	○	○
Linked In	○	○	○	○
You Tube	○	○	○	○
Google+	○	○	○	○
Pinterest	○	○	○	○
Tumblr	○	○	○	○
Instagram	○	○	○	○

If there are other social media services that you use, please list and rate your competence here.

7. How often do you use each of the following social media services?

	Never	Occasionally (not usually more than once a month	Regularly (not daily but at least weekly	Frequently (daily)	Very frequently (several times a day)
Facebook	○	○	○	○	○
Twitter	○	○	○	○	○
Linked In	○	○	○	○	○
You Tube	○	○	○	○	○
Google+	○	○	○	○	○
Pinterest	○	○	○	○	○
Tumblr	○	○	○	○	○
Instagram	○	○	○	○	○

If there are other social media services that you use, please list and rate how often you use them here

8. How important is each of the following reasons in encouraging you to use social media?

	Not at all important	Somewhat important	Very important
To stay in touch with current friends and family members	◯	◯	◯
To connect with old friends I have lost touch with	◯	◯	◯
To connect around a shared hobby	◯	◯	◯
To communicate about issues relating to medical training	◯	◯	◯

Not applicable, as I don't use social media

9. How important is each of the following factors in keeping you from using social media?

	Not at all important	Somewhat important	Very important
Lack of knowledge	◯	◯	◯
Lack of time	◯	◯	◯
Lack of interest	◯	◯	◯
Lack of perceived value	◯	◯	◯
Concern about harm to professional image	◯	◯	◯

Other (please specify)

10. Approximately how many hours a week do you spend using social media?

- ◯ Do not use
- ◯ Less than 1 hour
- ◯ 1-5 hours
- ◯ 6-10 hours
- ◯ 11-15 hours
- ◯ 16-20 hours
- ◯ More than 20 hours

EINSTEIN
Albert Einstein College of Medicine
OF YESHIVA UNIVERSITY

Einstein Medical Student Survey on Social Media

11. Approximately how many hours a week do you spend using the following social media services as part of your medical education?

	Do not use	Less than 1 hour	1-5 hours	6-10 hours	11-15 hours	16-20 hours	More than 20 hours
Facebook	◯	◯	◯	◯	◯	◯	◯
Twitter	◯	◯	◯	◯	◯	◯	◯
Linked In	◯	◯	◯	◯	◯	◯	◯
YouTube	◯	◯	◯	◯	◯	◯	◯
Google+	◯	◯	◯	◯	◯	◯	◯
Pinterest	◯	◯	◯	◯	◯	◯	◯
Tumblr	◯	◯	◯	◯	◯	◯	◯
Instagram	◯	◯	◯	◯	◯	◯	◯

12. How often do you monitor your online presence (e.g., Google yourself)?

Never	Occasionally (not usually more than once a month)	Regularly (not daily but at least weekly)	Frequently (daily)	Very frequently (several times a day)
◯	◯	◯	◯	◯

13. How often do you check Google Image for photos of yourself?

- ◯ Never
- ◯ Occasionally (not usually more than once a month)
- ◯ Regularly (not daily, but at least weekly)
- ◯ Frequently (daily)
- ◯ Very frequently (several times a day)

14. How important is each of the following reasons for monitoring your online presence?

	Not at all important	Somewhat important	Very important
To ensure that posted information is accurate	◯	◯	◯
To ensure that posted information is complete	◯	◯	◯
To ensure that posted information is professional	◯	◯	◯

Other (please specify)

15. What action have you taken if you find information that you believe should not be publicly available? Please check all answers that apply.

- ☐ Deleted people from my "friends" list
- ☐ Deleted comments made by others on my profile
- ☐ Removed my name from photos that were tagged to identify me
- ☐ I have not taken action.

Other (please specify)

16. Have you ever found that your online presence is:
Please check all answers that apply.

- ☐ Inaccurate
- ☐ Incomplete
- ☐ Unprofessional
- ☐ Absent

Other (please specify)

17. I use privacy settings.

- ◯ Yes
- ◯ No

18. I have accepted invitation(s) from patients to be "friends."

- ◯ Yes
- ◯ No

19. I have invited patients to be "friends."

- ◯ Yes
- ◯ No

20. I have accepted invitations by faculty to be "friends."

- ◯ Yes
- ◯ No

21. I have invited faculty to be "friends."

- ◯ Yes
- ◯ No

22. I have Googled faculty members.

- ◯ Yes
- ◯ No

23. Which of the following content have you posted online yourself?
Please check all answers that apply

- [ ] None
- [ ] Unidentifiable patient information
- [ ] Identifiable patient information
- [ ] Profanity
- [ ] Discriminatory language
- [ ] Depiction of intoxication
- [ ] Sexually suggestive material
- [ ] Items I thought were initially appropriate, but for various reasons later took down.

Other (please specify)

24. Which of the following types of information have you seen posted online by a classmate? Please check all answers that apply.

- [ ] None
- [ ] Unidentifiable patient information
- [ ] Identifiable patient information
- [ ] Use of profanity
- [ ] Depiction of intoxication
- [ ] Sexually suggestive material
- [ ] Items I found objectionable but did not discuss with my classmate
- [ ] Items I found objectionable and did discuss with my classmate

Other (please specify)

25. List the social media services you are aware of that provide benefit to patients.

26. When I become a physician, I intend to use social media to interact with my patients.

- Yes
- No
- Undecided

27. What concerns do you have about social media use?

	Not at all important	Somewhat important	Very important
Public perceptions of unprofessional behavior by me			
Family perceptions of unprofessional behavior by me			
Public perceptions of unprofessional behavior by my colleagues			
Public perceptions of my medical school			
Public perceptions of the medical profession			
Violations of patient confidentiality			
Posting of inaccurate medical information for patients			

Other (please specify)

28. To what extent do you agree or disagree with the following statement?
Patients use social media to obtain medical information.

- Strongly Agree
- Agree
- Disagree
- Strongly disagree

29. To what extent do you agree or disagree with the following statement?
The benefits of social media use in medicine outweigh its risks.

- Strongly agree
- Agree
- Disagree
- Strongly disagree

30. To what extent do you agree or disagree with the following statement?
As a student of medicine, it is my obligation to keep current on social media use.

- Strongly agree
- Agree
- Disagree
- Strongly Disagree

31. To what extent do you agree or disagree with the following statement?
Guiding patients online is a new responsibility for physicians in the digital age.

- Strongly Agree
- Agree
- Disagree
- Strongly disagree

32. Your personal data can be made accessible online without your deliberate intervention.

- True
- False

33. Einstein has a policy on social media usage.

- ○ True
- ○ False

34. Which medically-related blogs do you visit regularly?

35. Please add any additional comments about social media and medical professionalism here:

36. What additional information would you like to learn about social media?
