# Supplementary material for: Who’s misbehaving? Perceptions of unprofessional social media use by medical students and faculty
Source: BMC Med Educ. 2016 Feb 18;16:67. doi: 10.1186/s12909-016-0572-x (PMC4757980; doi:10.1186/s12909-016-0572-x)
Supplement: Additional file 2: — Einstein Faculty Survey on Social Media. (PDF 460 kb) [file 12909_2016_572_MOESM2_ESM.pdf]

EINSTEIN
Albert Einstein College of Medicine
OF YESHIVA UNIVERSITY

# Einstein Faculty Survey on Social Media

1. What is your gender?

- Male
- Female
- Other

2. What is your age?

- 25-29
- 30-39
- 40-49
- 50-59
- 60-69
- 70-79
- 80-

3. How do you identify yourself?

- White
- Black or African American
- American Indian or Alaska Native
- Asian Indian
- Pakistani
- Asian
- Native Hawaiian or other Pacific Islander
- Spanish/Hispanic/Latino/Latina

Other (please specify)

4. What is your specialty?

- ◯ Anesthesiology
- ◯ Emergency Medicine
- ◯ Family Medicine
- ◯ Geriatrics
- ◯ Internal Medicine
- ◯ Internal Medicine Subspecialty (exclusive of Geriatrics)
- ◯ Neurology
- ◯ Obstetrics/Gynecology
- ◯ Pediatrics
- ◯ Pediatrics Subspecialty
- ◯ Psychiatry
- ◯ Radiology
- ◯ Physical Medicine and Rehabilitation
- ◯ Surgery
- ◯ Surgical Subspecialty
- ◯ Basic Science Medical Educator (non-MD)
- ◯ Social Science Medical Educator (e.g., RN, CSW, PsyD, JD, etc.)
- ◯ Other

If "Other," please specify.

5. Approximately how much of your time at work do you spend teaching medical students, residents, or fellows?

- ◯ I do not teach at all
- ◯ Up to 25%
- ◯ 26-50%
- ◯ 51-75%
- ◯ 76-99%
- ◯ I teach 100% of the time

6. How familiar are you with each of the following social media services?

| | Never heard of it | Heard of it; not sure of its purpose | Familiar with its purpose | Very familiar with its purpose |
|---|---|---|---|---|
| Facebook | ○ | ○ | ○ | ○ |
| Twitter | ○ | ○ | ○ | ○ |
| Linked In | ○ | ○ | ○ | ○ |
| YouTube | ○ | ○ | ○ | ○ |
| Google+ | ○ | ○ | ○ | ○ |
| Pinterest | ○ | ○ | ○ | ○ |
| Tumblr | ○ | ○ | ○ | ○ |
| Instagram | ○ | ○ | ○ | ○ |

If there are other social media services with which you are familiar, please list and rate your familiarity here.

7. How competent are you at using each of the following social media services?

| | Not at all | Beginner | Competent | Highly competent |
|---|---|---|---|---|
| Facebook | ○ | ○ | ○ | ○ |
| Twitter | ○ | ○ | ○ | ○ |
| Linked In | ○ | ○ | ○ | ○ |
| YouTube | ○ | ○ | ○ | ○ |
| Google+ | ○ | ○ | ○ | ○ |
| Pinterest | ○ | ○ | ○ | ○ |
| Tumblr | ○ | ○ | ○ | ○ |
| Instagram | ○ | ○ | ○ | ○ |

If there are other social media services that you use, please list and rate your competence here.

8. How often do you use each of the following social media services?

| | Never | Occasionally (not usually more than once a month | Regularly (not daily but at least weekly | Frequently (daily) | Very frequently (several times a day) |
|---|---|---|---|---|---|
| Facebook | ◯ | ◯ | ◯ | ◯ | ◯ |
| Twitter | ◯ | ◯ | ◯ | ◯ | ◯ |
| Linked In | ◯ | ◯ | ◯ | ◯ | ◯ |
| YouTube | ◯ | ◯ | ◯ | ◯ | ◯ |
| Google+ | ◯ | ◯ | ◯ | ◯ | ◯ |
| Pinterest | ◯ | ◯ | ◯ | ◯ | ◯ |
| Tumblr | ◯ | ◯ | ◯ | ◯ | ◯ |
| Instagram | ◯ | ◯ | ◯ | ◯ | ◯ |

If there are other social media services that you use, please list and rate how often you use them here

9. How important is each of the following reasons in encouraging you to use social media?

| | Not at all important | Somewhat important | Very important |
|---|---|---|---|
| To stay in touch with current friends and family members | ◯ | ◯ | ◯ |
| To connect with old friends I have lost touch with | ◯ | ◯ | ◯ |
| To connect around a shared hobby | ◯ | ◯ | ◯ |
| To teach medical students, residents, or fellows | ◯ | ◯ | ◯ |
| To communicate with other medical educators about educational issues in medicine | ◯ | ◯ | ◯ |
| To communicate about issues relating to medical practice | ◯ | ◯ | ◯ |

Other (please specify)

10. How important is each of the following factors in keeping you from using in social media?

| | Not at all important | Somewhat important | Very important |
|---|---|---|---|
| Lack of knowledge | ○ | ○ | ○ |
| Lack of time | ○ | ○ | ○ |
| Lack of interest | ○ | ○ | ○ |
| Lack of perceived value | ○ | ○ | ○ |
| Concern about harm to professional image | ○ | ○ | ○ |

Other (please specify)

11. Approximately how many hours a week do you spend using social media?

- ○ Do not use
- ○ Less than 1 hour
- ○ 1-5 hours
- ○ 6-10 hours
- ○ 11-15 hours
- ○ 16-20 hours
- ○ More than 20 hours

12. Approximately how often do you use the following social media services in the context of medical education?

| | Never | Occasionally (in less than 25% of my teaching sessions) | Regularly (in 25-49% of my teaching sessions) | Frequently (in 50-74% of my teaching sessions) | Very Frequently (in 75-100% of my teaching sessions) |
|---|---|---|---|---|---|
| Facebook | ○ | ○ | ○ | ○ | ○ |
| Twitter | ○ | ○ | ○ | ○ | ○ |
| Linked In | ○ | ○ | ○ | ○ | ○ |
| You Tube | ○ | ○ | ○ | ○ | ○ |
| Google+ | ○ | ○ | ○ | ○ | ○ |
| Pinterest | ○ | ○ | ○ | ○ | ○ |
| Tumblr | ○ | ○ | ○ | ○ | ○ |
| Instagram | ○ | ○ | ○ | ○ | ○ |

If there are other social media services that you use in the context of medical education, please specify and estimate percentage here.

## Einstein Faculty Survey on Social Media

13. How often do you monitor your online presence (e.g., Google yourself)?

- Never
- Occasionally (not usually more than once a month)
- Regularly (not daily but at least weekly)
- Frequently (daily)
- Very frequently (several times a day)

14. How often do you check Google Image for photos of yourself?

- Never
- Occasionally (not usually more than once a month)
- Regularly (not daily, but at least weekly)
- Frequently (daily)
- Very frequently (several times a day)

15. How important is each of the following reasons for monitoring your online presence?

| | Not at all important | Somewhat important | Very important |
|---|---|---|---|
| To ensure that posted information is accurate | ○ | ○ | ○ |
| To ensure that posted information is complete | ○ | ○ | ○ |
| To ensure that posted information is professional | ○ | ○ | ○ |

Other (please specify)

16. What action have you taken if you find information that you believe should not be publicly available? Please check all answers that apply.

- [ ] Deleted people from my "friends" list
- [ ] Deleted comments made by others on my profile
- [ ] Removed my name from photos that were tagged to identify me
- [ ] I have not taken action.

Other (please specify)

17. Have you ever found that your online presence is:
Please check all answers that apply.

- [ ] Inaccurate
- [ ] Incomplete
- [ ] Unprofessional
- [ ] Absent

Other (please specify)

18. Please answer yes or no to the following questions regarding your use of social media services (Facebook, etc.):

| | Yes | No |
|---|---|---|
| I use privacy settings. | ○ | ○ |
| I have accepted invitations from patients to be "friends." | ○ | ○ |
| I have invited patients to be "friends." | ○ | ○ |
| I have accepted invitations by medical students to be "friends." | ○ | ○ |
| I have invited medical students to be "friends." | ○ | ○ |

19. I have Googled students.

○ Yes

○ No

20. Which of the following content have you posted online yourself?
Please check all answers that apply.

- [ ] None
- [ ] Unidentifiable patient information
- [ ] Identifiable patient information
- [ ] Profanity
- [ ] Discriminatory language
- [ ] Depiction of intoxication
- [ ] Sexually suggestive material
- [ ] Items I thought were initially appropriate, but for various reasons later took down

Other (please specify)

21. Which of the following types of information have you seen posted online by a colleague?
Please check all answers that apply.

- [ ] None
- [ ] Unidentifiable patient information
- [ ] Identifiable patient information
- [ ] Use of profanity
- [ ] Depiction of intoxication
- [ ] Sexually suggestive material
- [ ] Items I found objectionable but did not discuss with my colleague
- [ ] Items I found objectionable and did discuss with my colleague

Other (please specify)

EINSTEIN
Albert Einstein College of Medicine
OF YESHIVA UNIVERSITY

## Einstein Faculty Survey on Social Media

22. List the social media services you are aware of that provide benefit to patients.

23. I use social media to interact with my patients (not including e-mail).

- Yes
- No

If yes, please list here.

24. I use social media to discuss my work.

- Yes
- No

If yes, please list here.

25. What concerns do you have about social media use?

| | Not at all important | Somewhat important | Very important |
|---|---|---|---|
| Public perceptions of unprofessional behavior by me | ○ | ○ | ○ |
| Family perceptions of unprofessional behavior by me | ○ | ○ | ○ |
| Public perceptions of unprofessional behavior by my colleagues | ○ | ○ | ○ |
| Public perceptions of my medical school | ○ | ○ | ○ |
| Public perceptions of the medical profession | ○ | ○ | ○ |
| Violations of patient confidentiality | ○ | ○ | ○ |
| Posting of inaccurate medical information for patients | ○ | ○ | ○ |

Other (please specify)

26. To what extent do you agree or disagree with the following statement?

| | Strongly agree | Agree | Disagree | Strongly disagree |
|---|---|---|---|---|
| One of the responsibilities of a medical educator is to counsel students or trainees on the appropriate use of social media. | ◯ | ◯ | ◯ | ◯ |
| Patients use social media to obtain medical information. | ◯ | ◯ | ◯ | ◯ |
| The benefits of social media use outweigh the risks. | ◯ | ◯ | ◯ | ◯ |
| As a physician, it is my obligation to keep current on social media use. | ◯ | ◯ | ◯ | ◯ |
| Guiding patients online is a new responsibility for physicians in the digital age. | ◯ | ◯ | ◯ | ◯ |

27. Your personal data can be made accessible online without your deliberate intervention.

◯ True

◯ False

28. Einstein has a policy on social media usage.

◯ True

◯ False

29. Do you visit medically-related blogs?

◯ Yes

◯ No

If yes, please list here.

30. Have you ever written for a medically-related blog?

- ◯ Yes
- ◯ No

If yes, please list here.

31. Please add any additional comments about social media and medical professionalism here:

32. What additional information would you like to learn about social media?

33. Have you previously filled out this social media survey (sent by Marti Grayson and Elizabeth Kitsis)?

- ◯ Yes
- ◯ No
- ◯ Unsure
